# Neuroinflammation Induced by Surgery Does Not Impair the Reference Memory of Young Adult Mice

**DOI:** 10.1155/2016/3271579

**Published:** 2016-11-13

**Authors:** Yanhua Zhao, Lili Huang, Huan Xu, Guangxi Wu, Mengyi Zhu, Jie Tian, Hao Wang, Xiangrui Wang, Weifeng Yu, Liqun Yang, Diansan Su

**Affiliations:** ^1^Department of Anesthesiology, Renji Hospital, School of Medicine, Shanghai Jiaotong University, Shanghai, China; ^2^Department of Anesthesiology, Shanghai Pulmonary Hospital, Tongji University School of Medicine, Shanghai, China; ^3^Department of Anesthesiology, Shanghai Tenth People's Hospital, Shanghai, China; ^4^Shanghai Universities Collaborative Innovation Center for Translational Medicine, Shanghai, China

## Abstract

Postoperative cognitive dysfunction (POCD) increases morbidity and mortality after surgery. But the underlying mechanism is not clear yet. While age is now accepted as the top one risk factor for POCD, results from studies investigating postoperative cognitive functions in adults have been controversial, and data about the very young adult individuals are lacking. The present study investigated the spatial reference memory, IL-1*β*, IL-6, and microglia activation changes in the hippocampus in 2-month-old mice after anesthesia and surgery. We found that hippocampal IL-1*β* and IL-6 increased at 6 hours after surgery. Microglia were profoundly activated in the hippocampus 6 to 24 hours after surgery. However, no significant behavior changes were found in these mice. These results indicate that although anesthesia and surgery led to neuroinflammation, the latter was insufficient to impair the spatial reference memory of young adult mice.

## 1. Introduction

Postoperative cognitive dysfunction (POCD) is the deterioration of cognitive function, especially learning and memory, which may last for days, months, or even years [1–3]. POCD occurs after cardiac and noncardiac surgeries and increases first-year morbidity and mortality after surgery [4–6]. However, the mechanism of POCD is unclear yet.

There is no doubt that aged individuals are more likely to develop POCD [7–9]. However, for the adult subjects, there is no clear conclusion. Maze's group demonstrated that learning and memory were impaired after anesthesia and surgery in 3-4-month-old mice. Rosczyk et al. [10] demonstrated that there were no signs of neuroinflammation or cognition impairment after surgery in adult mice (4–6-month-old). Similarly, Wuri et al. [11] observed no learning or memory changes after partial hepatectomy in adult mice (4-month-old).

According to the work of Finlay and Darlington [12], mice after two months are considered adult. Accordingly, 3–6-month-old mice and 2-month-old mice are biologically equivalent to 30–40-year-old human and college freshmen, respectively [13]. While Maze's group investigated the learning and memory after anesthesia and surgery in 3-4-month-old mice, no data in younger adult mice, that is, 2-month-old, are available. Unfortunately, this is an age at which particular diseases will arise and may need surgeries, such as appendicitis, osteosarcoma, and leukemia. In the present study, we investigated whether the spatial reference memory of 2-month-old mice will be affected after anesthesia and surgery.

## 2. Methods

This study was approved by the Animal Care and Use Committee of Shanghai Jiao Tong University, School of Medicine. All animal procedures were performed in accordance with the National Institutes of Health (NIH) animal care guidelines.

### 2.1. Animals and Experimental Grouping

Two-month-old male C57BL/6J mice were provided by the Animal Research Center of Shanghai Jiaotong University, School of Medicine. The animals were housed in standard cages under controlled laboratory conditions (temperature of 22 ± 2°C, 12-hour light/12-hour dark cycle) with free access to regular rodent pellets and water. All mice were allowed to adapt to their new environment for 7 days before beginning the experiments.

Mice were randomly divided into three groups: naïve group, anesthesia group, and surgery group. Splenectomy [14] was performed with neuroleptic anesthesia (intraperitoneal injection of 200 *μ*g/kg fentanyl and 10 mg/kg droperidol, as reported previously [15, 16]) in the surgery group. For the splenectomy, a small incision was made in the left upper abdominal quadrant, and the spleen was mobilized, isolated, and removed. The wound was infiltrated with 0.25% bupivacaine and closed by suture. A single dose of butorphanol (0.4 mg/kg, s.c.) was administered for postoperative analgesia at the end of surgery. An identical neuroleptic anesthesia regimen was administered to the mice in the anesthesia group. No interventions were conducted on the mice in the naïve group.

### 2.2. Morris Water Maze (MWM)

**Figure 1:**
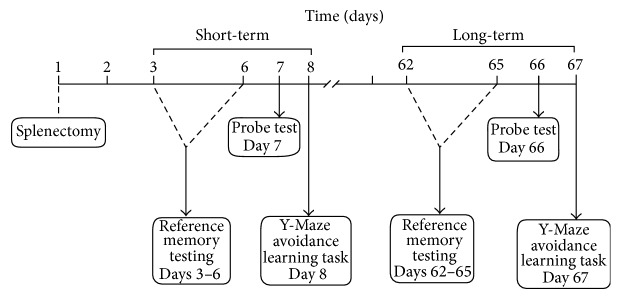
Schematic timeline of the experimental paradigm.

Spatial reference memory was evaluated in the MWM using a computerized video tracking system (days 3–7 and days 62–66 in Figure 1, *n* = 15). The reference memory test was performed on four days (days 3–6 and days 62–65 in Figure 1). For the reference memory, animals were maintained in the same rearing conditions throughout the test. The test was performed by an operator blinded to the group conditions. Briefly, a hidden round platform was placed 1 cm below the water surface and located in the center of the northeast quadrant in a circular pool (110 cm in diameter and 30 cm in depth). The water was maintained at 23–25°C, and the pool was situated in a room with visual cues. The position of the cues remained unchanged throughout the task.

In all the trials, each mouse was released into the water facing the pool wall from one of four separate quadrants and allowed to swim until it landed on the platform. Once the mouse found the platform, the trial was terminated, and the mouse was allowed to stay on the platform for 15 s. If the mouse failed to find the platform within 60 s, it was gently guided to the platform and allowed to remain on the platform for 15 s. Four trials were conducted per day, separated by a 5-minute intertrial interval, and the platform remained at the same location throughout the test. The amount of time spent finding and mounting the platform (escape latency) and the swimming speed were calculated from the recorded videos using MWM software (Shanghai Jiliang Software Technology Co. Ltd., China).

The probe test was performed on the day after the reference memory test (day 7 and day 66 in Figure 1). In this test, the platform was absent, and the animals were allowed to swim freely for 60 s, starting from the quadrant opposite the platform. The times spent in the target and opposite quadrant were recorded.

### 2.3. Y-Maze

Seven days or 66 days after surgery (day 8 and day 67 in Figure 1, *n* = 15), the avoidance learning task was performed as previously described [17, 18] in a Y-Maze equipped with electric grids in the floor. The grids were controlled by a computer, and a camera on the top of the Y-Maze recorded and provided the position of the animals to the computer. Of the three arms, one was defined as the “start arm,” another was the “wrong arm,” and the third was the “correct arm.” The animals had to leave the “start arm” within 5 seconds and escape into the “correct arm” to avoid foot shocks. Active avoidance errors were recorded if the animals did not leave the “start arm” within 5 seconds. If the mouse chose the “wrong arm,” a discrimination error was recorded. Foot shocks were administered for 7 seconds each until the animals chose the “correct arm.” The foot shock level was changed individually (maximum: 40 V) according to the performance of the mouse in the first trial or until the mouse suddenly lifted one or two paws from the grid at the bottom of the Y-Maze after the shock. One trial per minute was performed until the mouse reached the final criterion of correctly performing seven out of eight consecutive trials. In order to avoid odor confounding, the Y-Maze was cleaned by alcohol after each mouse finished its tasks. The numbers of total trials, active avoidance errors, and discrimination errors were recorded.

### 2.4. ELISA Assay for IL-1*β* and IL-6

Mice in each group were sacrificed 2 h, 6 h, 24 h, and 48 h after surgery. The mice were rapidly decapitated, and the brains were quickly removed. Hippocampus dissections were performed on ice-cold frosted glass, and tissues were quickly frozen in liquid nitrogen. The brain samples were stored at −80°C until analysis. The mouse hippocampus was homogenized in sterile 0.1 M PBS containing a complete protease inhibitor cocktail (Roche). The homogenates were centrifuged at 10,000 rpm for 15 min at 4°C, and the supernatants were analyzed for IL-1*β* and IL-6 using ELISA kit (R&D Systems, Minneapolis, MN). The protein concentrations of all samples were measured using a BCA protein assay kit (Pierce). The cytokine levels were expressed as fold change to naïve.

### 2.5. Activation of Microglia

For immunohistochemical analysis of microglia, all mice (*n* = 4) were deeply anesthetized using Equithesin [1% pentobarbital/4% (v/v) chloral hydrate; 3.5 mL/kg, i.p.] and perfused intracardially with saline followed by 4% paraformaldehyde in 0.1 M phosphate buffer (PB, pH 7.4). Brains were then harvested, postfixed in the same fixative for 4 hours at 4°C, and immersed in 10–30% gradient sucrose in PB for 24–48 hours at 4°C for cryoprotection. Brain tissue was freeze-mounted in OCT embedding medium, and 16 *μ*m thick coronal sections of hippocampus were cut sequentially and mounted on Superfrost Plus slides. Slices were permeabilized in 0.4% Triton X-100, blocked with 5% bovine serum albumin in 0.1% Triton X-100, and incubated overnight at 4°C with mouse anti-CD11b (Abcam, Cambridge, UK, 1 : 100). After rinsing in 0.1% Triton X-100 in PBS, sections were incubated with secondary antibodies conjugated with Alexa Fluor 488 (1 : 500; Invitrogen; Paisley, UK) for 1 hour in the dark. The adjacent sections were used as negative controls. All the procedures for negative controls were processed in the same manner except omitting primary antibody. Images were acquired with a Leica TCS SP2 confocal laser scanning microscope. ImageJ was used for the quantification of the positive percentage.

### 2.6. Statistical Analysis

All of the data are presented as mean ± SEM. The Statistical Package for the Social Sciences (SPSS) v.20.0 was used for the statistical analyses. Two-way ANOVA with repeated measures was used to analyze the water maze escape latency and average speed. One-way ANOVA was used for the probe quadrant trial data, avoidance learning task data, and the IL-1*β* and IL-6, followed by* post hoc* Bonferroni correction. Differences were considered to be statistically significant at *p* < 0.05.

## 3. Results

### 3.1. Cytokines Levels in the Hippocampus Increased Temporarily after Surgery

**Figure 2:**
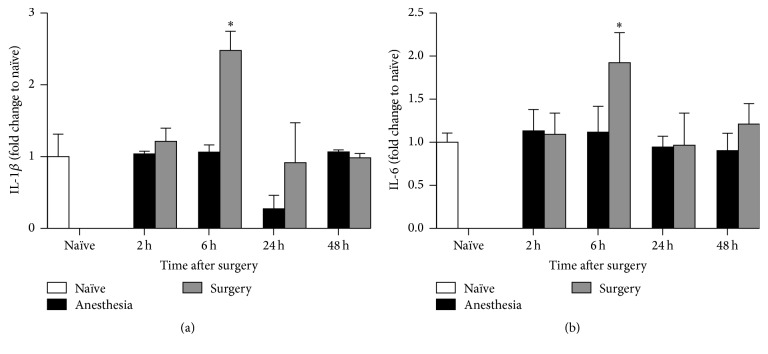
IL-1*β* and IL-6 levels in the hippocampus. Six hours after surgery, the hippocampal IL-1*β* and IL-6 levels increased significantly compared with those in the naïve group (*p* < 0.05). No significant differences were observed at other time points. ^*∗*^ *p* < 0.05 compared with the naïve group. For IL-1*β*, *n* = 9 in naïve group; *n* = 4 in 2 h, 6 h, and 48 h; *n* = 6 in 24 h for the anesthesia group. For IL-6, *n* = 10 in naïve group; *n* = 4 in 2 h, 6 h, and 48 h; *n* = 5 in 24 h for the surgery group.

Splenectomy significantly induced IL-1*β* and IL-6 expression in the hippocampus. Upregulation of these two cytokines was observed at 6 hours after surgery and decreased again by 24 hours postoperatively (Figures 2(a) and 2(b)). Anesthesia* per se* did not affect the level of IL-1*β* or IL-6 at any time point compared with the naïve controls.

### 3.2. Microglia Was Activated after Surgery

**Figure 3:**
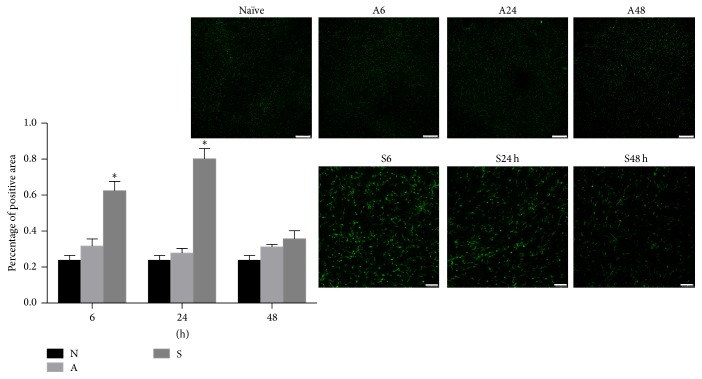
Microglia activation after surgery. Low levels of CD11b immunoreactivity were detected in the naïve and anesthesia groups at any time points studied. In contrast, an intensive number of CD11b positive cells were detected in the hippocampus at 6 h and 24 h after surgery in the surgery group. At 48 h after surgery, the proportion of CD11b positive cells was comparable in the surgery group and anesthesia group. The percentage of positive area was measured by ImageJ. Compared with the naïve group, it increased significantly in the surgery group in the 6 and 24 hours after surgery. N: naïve group; A: anesthesia group; S: surgery group. ^*∗*^ *p* < 0.05 compared with the naïve group

CD11b immunostaining showed that very few microglia were activated in the hippocampus from mice of naïve or anesthesia groups (Figure 3). On the contrary, surgery profoundly induced microglia activation in the hippocampus, which was intensive at 6 and 24 hours after surgery but started to decline by 48 hours (Figure 3).

### 3.3. Anesthesia and Surgery Have No Effects on the Short-Term and Long-Term Memory Ability in the Young Adult Mice

**Figure 4:**
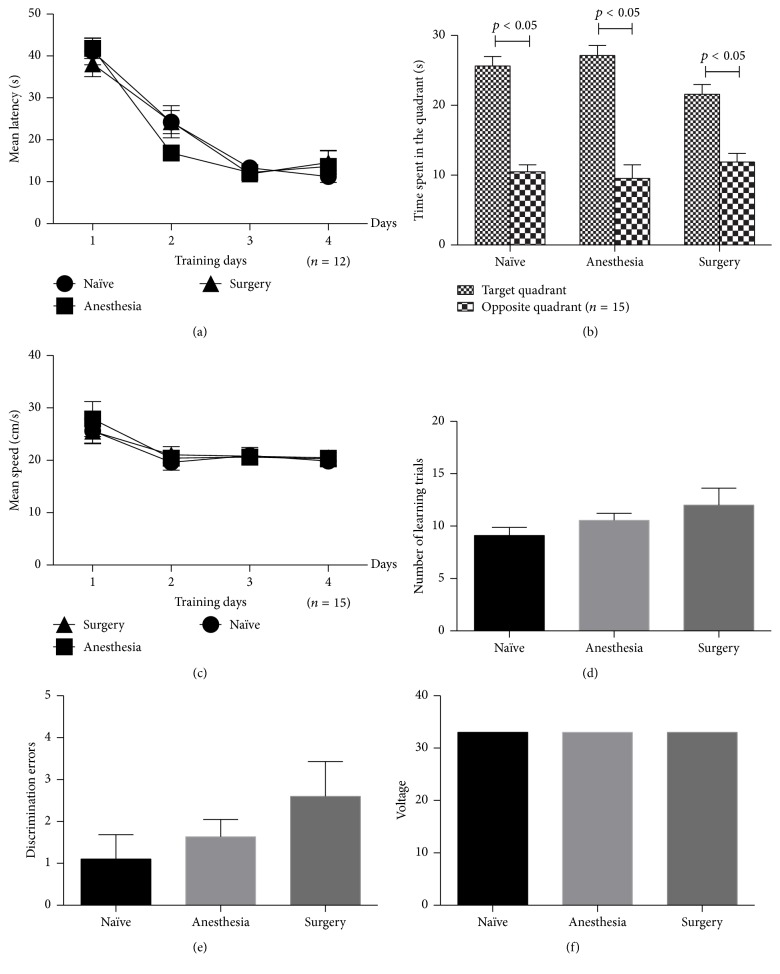
Morris water maze and avoidance learning task test results of short term. No significant differences were observed among the three groups. (a) Mean latency; (b) swimming time in the target quadrant; (c) average swimming speed; (d) the number of learning trials; (e) discrimination errors; (f) shock voltage (*n* = 15).

**Figure 5:**
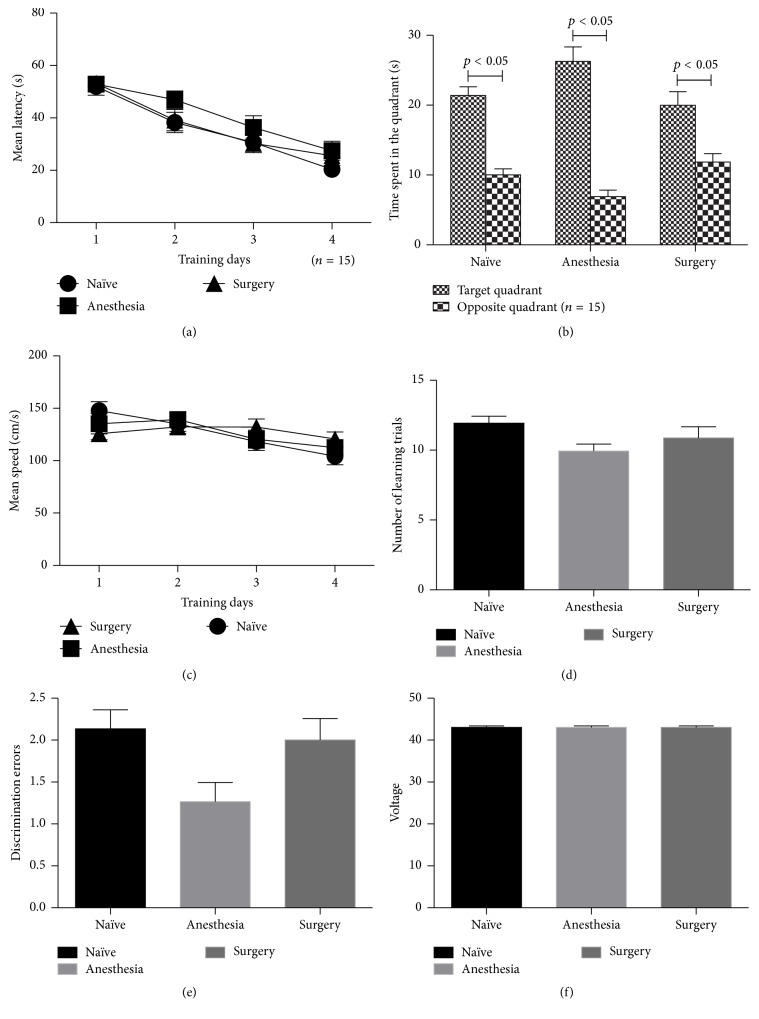
Morris water maze and avoidance learning task test results of long term. No significant differences were observed among the three groups. (a) Mean latency; (b) swimming time in the target quadrant; (c) average swimming speed; (d) the number of learning trials; (e) discrimination errors; (f) shock voltage (*n* = 15).

The results of the Morris water maze performed two days and two months after surgery revealed no differences among the three groups in latency, swimming speed, or swimming time in the target quadrant during the probe test (Figures 4 and 5). Similarly, no significant differences were observed in the results of the avoidance learning task in the Y-Maze, regardless of the number of learning trials, avoidance errors, or discrimination errors or voltage (Figures 4 and 5).

## 4. Discussion

We demonstrated in the present study that (a) anesthesia and surgery but not anesthesia only temporally increased hippocampal IL-1*β* and IL-6, as well as microglia activation in the hippocampus in 2-month-old young adult mice and that (b) anesthesia and surgery cannot hurt the short-term and long-term reference memory of such aged mice. These findings indicate that central inflammation induced by surgery does not necessarily lead to reference memory impairment in young adult mice.

Our present results are consistent with previous studies demonstrating that neuroinflammation can be induced by surgery. Wan et al. [15] reported elevated levels of IL-1*β* in the hippocampus after splenectomy in rats. Kálmán et al. [19] demonstrated that the concentration of IL-6 in cerebrospinal fluid significantly increased 1 week after cardiac surgery. Maze et al. [11, 20, 21] demonstrated that serum TNF-*α* induced by surgery disturbed the blood-brain barrier (BBB), which then stimulated macrophage migration into the hippocampus and promoted hippocampal neuroinflammation. We assume that the increases of IL-1*β* and IL-6 in hippocampus after surgery in the present study may arise through the same pathway, but more studies are needed to confirm the assumption in the young adult mice.

Other proinflammatory cytokines may also be involved in the development of POCD in aged animals. Ma et al. [22] demonstrated that TNF-*α*, IL-1*β*, IL-4, and IL-6 in the hippocampus increased after surgery and the TNF-*α* receptor antagonist attenuated the elevation of these cytokines. Wang et al. [23] found that the hippocampal IL-1*β*, TNF-*α*, and IFN-*γ* were overexpressed after surgery in aged mice. Hovens et al. [24] demonstrated that the hippocampal IL-6, IL-12, and IL1*β* increased after surgery in aged rats. More studies should be done to investigate whether such proinflammatory cytokines changed after surgery in young adult animals.

However, the neuroinflammation detected in the current study is insufficient to cause learning or memory impairment in such young adult mice, as we did not observe any short- or long-term changes in these aspects after surgery. We postulate that this is because the immune system in young adult mice is very strong so that the anti-inflammatory pathways, such as the vagus nerve pathway [21], are rapidly activated to reduce the inflammation.

In many previous experiments reporting impaired learning or memory ability after surgery, at least one other contributing factor exists. For example, Fidalgo et al. [25] observed impaired learning and memory after surgery in adult mice when they were simultaneously infected with LPS (50 ng/kg, a subclinical dose). The surgery-induced learning and memory impairment demonstrated by He et al. [26], Wan et al. [27], and Cao et al. [28] all happened in aged rats. Together, results from current and previous findings all indicate that other factors, such as old age or subclinical infection, are required so that the neuroinflammation induced by surgery would be deleterious enough to alter spatial reference memory in mice.

Morris water maze and active avoidance test were used in the present study to measure the spatial reference memory changes after anesthesia and surgery. Spatial reference memory is the ability to remember the relevance of spaces, which is a relative long-term memory compared with working memory. Working memory is a limited capacity that is responsible for the transient holding, processing, and manipulation of information, which is a relative short-term memory [29]. More studies should be done to investigate the changes of working memory after anesthesia and surgery.

Age is probably one of the key determinants of the responses to anesthesia and surgery. As most previous studies utilized 4–6-month-old mice [15, 20, 21, 30], the present study used for the first time two-month-old mice, which are biologically equivalent to 18-year-old human. Differences in ages among our study and previous studies are very possibly the reason for the different behavior tests results.

Since the spleen is an immunoregulatory organ and splenectomy may deteriorate the immunological system and therefore lead to an exaggerated inflammatory status [31], we are unable to rule out the possibility that the absence of spleen also contributed to the development of neuroinflammation. Other surgical procedures are needed to further determine the role of surgical trauma in the neuroinflammation and the following learning and memory changes.

## 5. Conclusions

Anesthesia and surgery lead to neuroinflammation. However, such neuroinflammation is insufficient to impair the spatial reference memory of young adult mice.
